# Nanoengineered γ MnO_2_ Accelerates the Degradation of Antibiotic-Resistant Biofilms

**DOI:** 10.3390/life16030367

**Published:** 2026-02-24

**Authors:** Moorthy Maruthapandi, Arulappan Durairaj, Gila Jacobi, Sivan Shoshani, Ehud Banin, John H. T. Luong, Aharon Gedanken

**Affiliations:** 1 Department of Chemistry, Bar-Ilan University, Ramat-Gan 52900, Israel; chemdraj@gmail.com; 2 Bar-Ilan Institute for Nanotechnology and Advanced Material, Bar-Ilan University, Ramat-Gan 52900, Israelsivan.shoshani@biu.ac.il (S.S.);; 3 School of Chemistry, University College Cork, T12 YN60 Cork, Ireland; luongprof@gmail.com

**Keywords:** MnO_2_ nanoparticles, ultrasonication, mechanical penetration, biofilm eradication, reactive oxygen species (ROS)

## Abstract

Bacterial biofilms remain a major challenge in clinical infections due to their dense extracellular polymeric substance (EPS) matrix and strong resistance to conventional antibiotics. This study reports manganese dioxide (MnO_2_) nanoparticles capable of autonomous navigation toward bacterial clusters, mechanical penetration of biofilm structures, redox-driven membrane disruption, and synergistic oxidative stress. The nanoparticles exhibit directional movement attributed to a combination of negatively charged surface potential, asymmetric topology, and catalytic reactivity toward bacterial metabolites. MnO_2_ demonstrates potent antibiofilm activity against MRSA and MDR *E. coli* (>98% eradication) and partial activity against Pseudomonas aeruginosa. Time-lapse microscopy, EPR spectroscopy, XPS analysis, and SEM imaging reveal that MnO_2_ disrupts both EPS and cell membranes while maintaining structural integrity throughout treatment. Cytotoxicity assays confirm ≥85% viability in human fibroblasts and keratinocytes at therapeutic concentrations. MnO_2_ shows controlled biodegradation into Mn^2+^ ions, which participate in physiological pathways and undergo renal clearance. These findings support MnO_2_ nanoparticles as promising biofilm-targeting agents for topical formulations, wound care, and implant coatings.

## 1. Introduction

As a global threat to traditional antibiotic therapy, bacterial biofilms have been attributed to 80% of infections in individuals [1]. The exopolysaccharide-covered surface-related bacterial development becomes resistant to chemical attacks and other severe environments, such as extreme pH and temperature [2,3]. The bacterial matrix enables bacteria to undergo numerous flexible modifications, resulting in over 1000-fold resistance to standard antimicrobial therapy and the host immune system [4,5]. Biofilms are crucial targets for biorthogonal treatments, as they persist in forming wounds, urine catheters, oral implants, and other indwelling medical devices [6,7]. The spreading of the hydrophobic molecule is purposefully delayed and strengthened by the biofilm matrix, making therapy largely challenging [8]. Microbial cells embedded in extracellular networks become highly resistant to harsh environmental conditions, antibiotics [9,10], and host immune response systems. The treatment of infections associated with biofilms is very difficult and ineffective in wounds and indwelling therapeutic devices [11]. The resulting prolonged use of antibiotics for treating those illnesses also contributes to the formation of antibiotic-resistant strains.

Metal oxide (MO) nanoparticles (NPs) with unique physicochemical properties exhibit remarkable antibacterial and antifungal properties and are effective against multidrug-resistant (MDR) bacteria and various fungal pathogens [12]. They display targeted interactions with biofilm matrices and generate reactive oxygen species (ROS) to disrupt bacterial cell walls and other important biomolecules. MO NPs, including ZnO, CuO, TiO_2_, Fe_3_O_4_, MgO, and doped metal oxides, are stable, biocompatible, and have facile surface functionalization [13,14]. Based on their intrinsic antimicrobial properties, MO NPs are effective against biofilm-forming bacteria [15]. Therefore, single-component metal oxide nanoparticles will be a promising frontier in addressing antibiotic resistance and biofilm-associated infections, opening new avenues for research and clinical applications [16]. However, they suffer from long-term toxicity, environmental impact, and potential resistance development even after doping, functionalization, and heterojunction.

MnO_2_ NPs are inexpensive, eco-friendly, and highly reactive with diverse morphologies and biocompatibility. These emerging materials have several applications in medicine, ion exchange, catalysis, sensing, adsorption, and the energy domain [17]. Their versatile antibacterial and antibiofilm activities are due to multiple oxidation states and a large surface area to interact strongly with bacterial cell membranes via combined forces [18]. Of note, MnO_2_ NPs induce the reactive oxygen species (ROS), causing oxidative stress to damage the bacterial cell membrane [19]. This study describes the synthesis of MnO_2_ NPs and their effective removal of antibiotic-resistant biofilms of MRSA and MDR *E. coli*, two harmful pathogens of high WHO concern. The MnO_2_ nanocomposite exhibits autonomous navigation toward bacterial cells through a combination of electrostatic attraction arising from its negative zeta potential, high surface reactivity that promotes catalytic interactions with bacterial metabolites, and asymmetric nanoparticle morphology that enhances mobility within the complex extracellular polymeric substance (EPS) environment. Time-lapse microscopy confirmed that MnO_2_ actively migrates toward dense bacterial clusters, supporting its biofilm-targeting capability. Fully characterized MnO_2_ NPs are effective against mature biofilms because they release reactive oxygen species (ROS) and absorb energy from the environment for self-propulsion.

## 2. Material and Methods

All chemicals (Manganese sulfate and ammonium persulfate (APS)) with the highest purity were purchased from Sigma-Aldrich, Jerusalem, Israel.

### 2.1. Materials

MnO_2_ hierarchical microspheres were synthesized via a green ultrasonic method followed by a facile hydrothermal process. In a typical procedure, 8 mmol of manganese sulfate was dissolved in 40 mL of deionized water under continuous magnetic stirring to obtain a homogeneous solution. Subsequently, 8 mmol of ammonium persulfate was added to the solution, followed by ultrasonic irradiation for 30 min. During the ultrasonic irradiation process, MnO_2_ nuclei were formed. The resulting solution was then transferred into a Teflon-lined stainless-steel autoclave and heated at 80 °C for 10 h in an electric oven, after which it was allowed to cool naturally to room temperature. During the hydrothermal process, Mn atoms self-assembled into hierarchical MnO_2_ microspherical structures. Upon completion of the reaction, the dark black precipitate was collected by centrifugation and washed several times with deionized water and ethanol to remove residual ions and impurities. The obtained product was finally dried at 60 °C for 12 h to yield MnO_2_ hierarchical microspheres.

### 2.2. Biofilm Eradication

For the biofilm eradication experiments, multi-drug-resistant *S. aureus* ATCC (American Type Culture Collection) 43300, *E. coli* MDR ATCC 2452, and *P. aeruginosa* (laboratory strain) were grown overnight in Mueller Hinton (MH) broth at 37 °C with shaking at 250 RPM. The overnight cultures were then diluted in 1% MH medium to an optical density (OD) of 0.01 for *S. aureus*, 0.3 for *E. coli*, and 0.05 for *P. aeruginosa*. For *S. aureus*, the medium was supplemented with 0.2% glucose. Glass slides (1 cm × 1.3 cm) were placed at the bottom of a 12-well plate, and 1 mL of the bacterial suspension was added to each well. The plates were incubated overnight at 37 °C for approximately 18 h to allow biofilm formation. The following day, the samples were rinsed three times with sterile double-distilled water (DDW) and transferred to a new plate. Then, 0.5 mL of 2% MH medium was added to each well, followed by 0.5 mL of either DDW (as a control) and MnO_2_ solution, and incubated again overnight at 37 °C for approximately 18 h. Afterwards, the samples were rinsed three times with sterile DDW. The biofilm formed on each glass slide was scraped off and suspended in 0.25 mL of fresh medium, from which samples were taken for serial dilution and viable cell counting [20].

### 2.3. Biocompatibility Assays

% Cell viability = (OD_sample_ − OD_blank_)/(OD_control_ − OD_blank_) × 100

OD_sample_ = Absorbance of NPs containing cell suspension, OD_blank_ = Absorbance of DMEM, and OD_control_ = Absorbance of cell suspension.

## 3. Instrumentation

Morphological studies of MnO_2_ were conducted using transmission electron microscopy (TEM, JEOL-2100, Peabody, MA, USA). A drop of MnO_2_ suspension was placed on a copper grid and dried overnight at 60 °C. Crystalline structure and elemental composition analyses were carried out using X-ray diffraction (XRD, Bruker AXS D8 Advance diffractometer, Berlin, Germany) and X-ray photoelectron spectroscopy (XPS, Nexsa spectrometer, Cambridge, UK). Bacterial morphology before and after treatment with MnO_2_ was examined using high-resolution scanning electron microscopy (HR-SEM). The mechanistic study of MnO_2_ activity against various bacteria was further supported by electron paramagnetic resonance (EPR) spectroscopy. Morphological characterization, bacterial cell penetration, and live-cell imaging were performed using HR-SEM (Magellan 400 L, FEI–Thermo Fisher Scientific, Waltham, MA, USA) operated at 5 kV.

## 4. Result and Discussion

**Figure 1:**
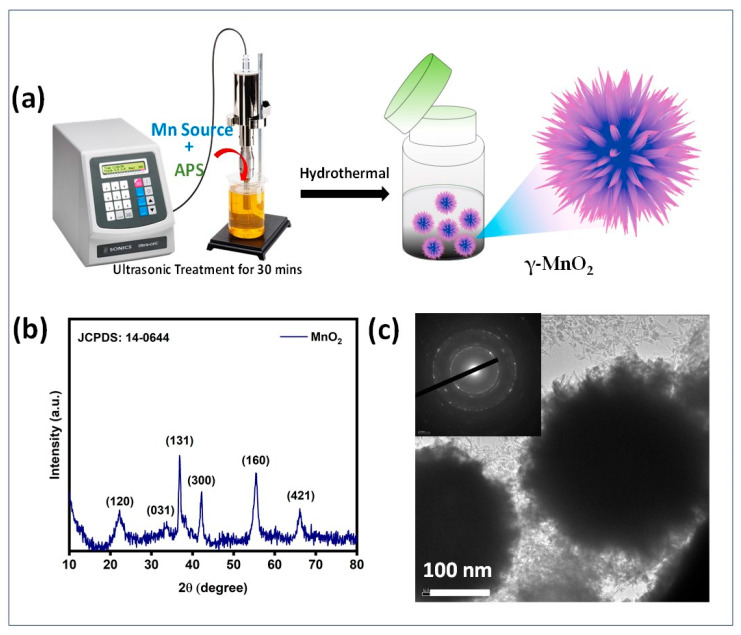
(**a**) Schematic presentations of the synthesis of MnO_2_ NPs, (**b**) XRD data of MnO_2_, and (**c**) TEM image of the MnO_2_ (inset: SAED pattern).

**Figure 2:**
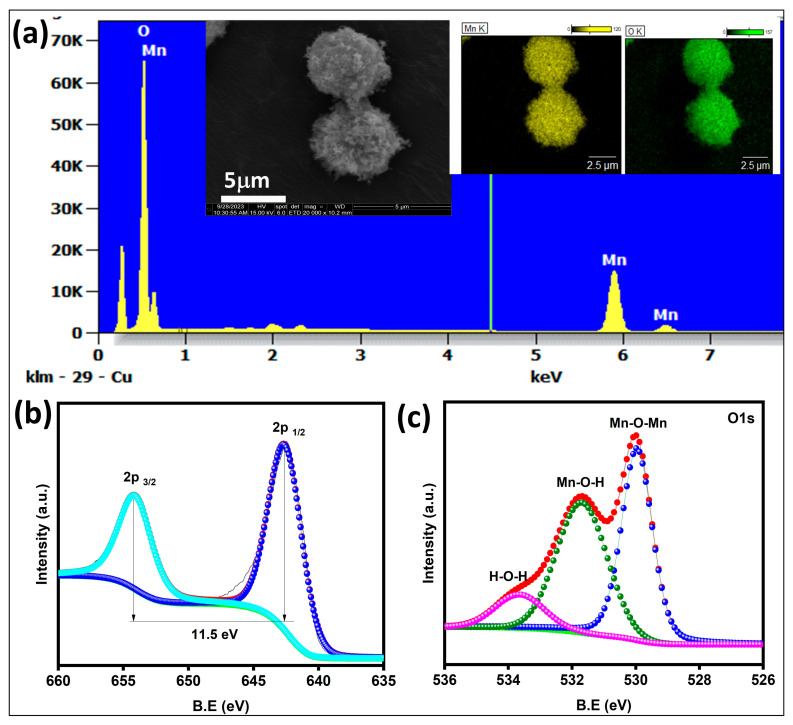
(**a**) Energy dispersive spectrum of the MnO_2_ NPs (Inset SEM image and Elemental mapping of Mn and Oxygen elements), (**b**) The XPS deconvoluted spectrum of Mn2p, and (**c**) XPS deconvoluted spectrum of O1s.

The MnO_2_ particles were successfully prepared by a controllable ultrasonic method followed by a hydrothermal technique. In this process, APS acts as an oxidant, which converts Mn^2+^ into the Mn^4+^ oxidation state [21]. Ultrasonic irradiation enhances the rapid nucleation and anisotropic growth, which leads to the formation of hierarchical nanoflower MnO_2_. The zeta potential of MnO_2_ nanoparticles was determined to be −6.81 mV by dynamic light scattering (DLS). The structure and crystal phase composition of manganese oxide samples prepared were examined by wide-angle powder XRD (Figure 1b). The diffraction peaks located at 21, 36,37,41,55, and 65⸰ were assigned to the planes (120), (031), (131), (300), (160), and (421) respectively, indicating the presence of γ-MnO_2_. The XRD pattern of the MnO_2_ matched the standard XRD of urchin-like manganese oxide crystal (JCPDS-14-0644) [22]. No other phases of MnO_2_ and impurities were observed, and MnO_2_ exhibits a monodispersed sphere-like structure (Figure 1c). The selected area electron diffraction (SAED) pattern shows several diffraction rings, corresponding to the (120), (131), (300), (160), and (421) planes of the MnO_2_ (JCPDS No: 14-0644) [22]. The EDS analysis indicates that the presence of Mn and oxygen elements indicates the formation of MnO_2_ NPs without any impurities (Figure 1a). The morphological properties of the synthesized MnO_2_ were characterized by scanning electron microscopy (SEM). The MnO_2_ sample exhibits monodispersed three-dimensional (3D) microscopic spheres with average sizes of about 4 μm (Figure 1a inset). Elemental mapping and EDAX were conducted to analyze the elemental composition of sphere-like MnO_2_ NPs (Figure 1a inset). From the elemental distribution images, the manganese and oxygen elements were dispersed uniformly across the selected area of the MnO_2_. The surface functional groups and elemental compositions of MnO_2_ NPs were investigated using X-ray photoelectron spectroscopy (XPS), as shown in Figure 2b,c. The high-resolution Mn2p signals of MnO_2_ were deconvoluted into two peaks at 641 eV and 654 eV, corresponding to the Mn 2p_3/2_ and Mn 2p_1/2,_ respectively [23]. The spin energy separation between Mn 2p_3/2_ and Mn 2p_1/2_ was 11.6 eV, which proves the presence of Mn^4+^ valence in MnO_2_. The high resolution of O1s peaks deconvoluted into three peaks at 529.8, 532, and 534 eV, which correspond to the presence of Mn-O-Mn, Mn-O-H, and adsorbed water molecules, respectively [24].

### 4.1. Biofilm Eradication

**Figure 3:**
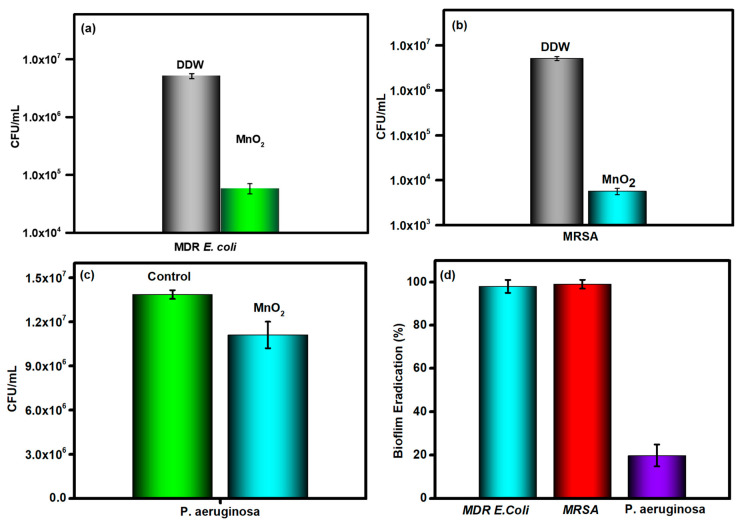
(**a**–**c**) Biofilm inhibition ability of MnO_2_ against *E. coli*, MRSA, and *P. aeruginosa*; (**d**) The percent biofilm inhibition of MDR *E. coli*, MRSA, and *P. aeruginosa* biofilm by MnO_2_ nanoparticles.

The biofilm eradication property of MnO_2_ was studied against three antibiotic-resistant bacteria: *E. coli* MDR, MRSA, and PA. Following 18 h of biofilm treatment by MnO_2_ NPs, marked differences were observed among these test organisms. For MDR *E. coli* and MRSA, the biofilm subjected to MnO_2_ NPs significantly reduced cell counts, compared to the control. The reduction in CFU indicates a substantial disruption of biofilm mass as well as bacterial survival (Figure 3a,b); however, the PA biofilm remained intact (Figure 3c), revealing the relative resistance of PA biofilms to MnO_2_-mediated eradication. The MnO_2_ NPs display substantial antibiofilm efficacy against MDR *E. coli* and MRSA but have limited to no effect on PA. MnO_2_ nanoparticles gradually convert into Mn^2+^ ions under mildly acidic or reducing conditions typical of infected tissues. Released Mn^2+^ can participate in physiological enzymatic pathways and is cleared renally. This biodegradation pathway supports the biocompatibility and long-term safety of MnO_2_-based antimicrobial formulations.

Biofilms function as a physical shield against the attack of MnO_2_ NPs and reduce the metabolic rates of their inhabiting cells under harsh environments. In this study, the biofilm destruction of MnO_2_ NPs was strain dependent as they were very effective against MDR *E. coli* and MRSA, but moderately effective against PA. In MRSA, the absence of an outer layer and less complex extracellular polymeric properties cannot prevent the binding of MnO_2_ NPs/their reactive intermediates to the bacterial membranes, resulting in the disturbance of biofilm structure. The outer membrane of MDR *E. coli* is also effective in preventing the penetration of MnO_2_ NPs. In contrast, the *Pseudomonas* biofilm consists of a large amount of extracellular polymeric substances (EPSs), which form a diffusion barrier to prevent the diffusion of antimicrobial agents. Biofilms also serve to trap MnO_2_ NPs and prevent their binding of MnO_2_ NPs to the PA membrane. Such a result also suggests that MnO_2_ NPs were not effective in hydrolyzing the *Pseudomonas* film, which consists of diverse biomolecules, including antioxidant enzymes that effectively neutralize the ROS released by MnO_2_ NPs. The effective antibiofilm behavior observed against MDR *E. coli* and MRSA demonstrates the potential utility of MnO_2_ NPs in preventing and treating biofilm-related infections, particularly in implanted medical devices. Nevertheless, the negligible effect of MnO_2_ NPs against PA is a major concern, as this bacterium is often associated with chronic, intractable biofilm infections such as those in cystic fibrosis and wound settings.

MnO_2_ NPs exhibit multiple mechanisms to act on several bacterial targets, whereas traditional antibiotics are designed to target a single microbial biomolecule. First, MnO_2_ NPs interact with the bacterial membrane through non-covalent forces, hydrophobic interactions, van der Waals forces, and receptor–ligand interactions. The strong attachment of MnO_2_ NPs to bacterial surface components interferes with important bacterial functions to support their growth and survival. Second, the generation of ROS is very detrimental to bacteria because ROS are known to disrupt metabolic processes, inhibit respiratory enzymes and induce oxidative stress, resulting in membrane disruption and leakage of cell contents [25]. Notably, the dissolution of MnO_2_ NPs releases Mn^2+^ ions that interact with sulfhydryl (–SH) groups of bacterial proteins to form stable S—Mn bonds, resulting in protein inactivation, disruption of membrane permeability, and subsequent cell lysis [26]. Mn^2+^ ions in contact with bacterial cell membranes reduce their dipole potential, alter the hydration state of the phospholipid headgroups as well as the net surface charge, causing local disruption of the membrane structure and increasing permeability [27]. ROS generated by Mn-based OA-MnO_2_ nanozymes effectively eradicate Gram-positive *S. aureus* and Gram-negative *E. coli* [28]. Mo/Cs-doped MnO_2_ also exhibits antibacterial efficacy against *S. aureus* and *E. coli* [29]. The treatment of a bacterial population with MnO_2_ under dark conditions destroys bacterial lipid molecules and cell walls [30].

**Figure 4:**
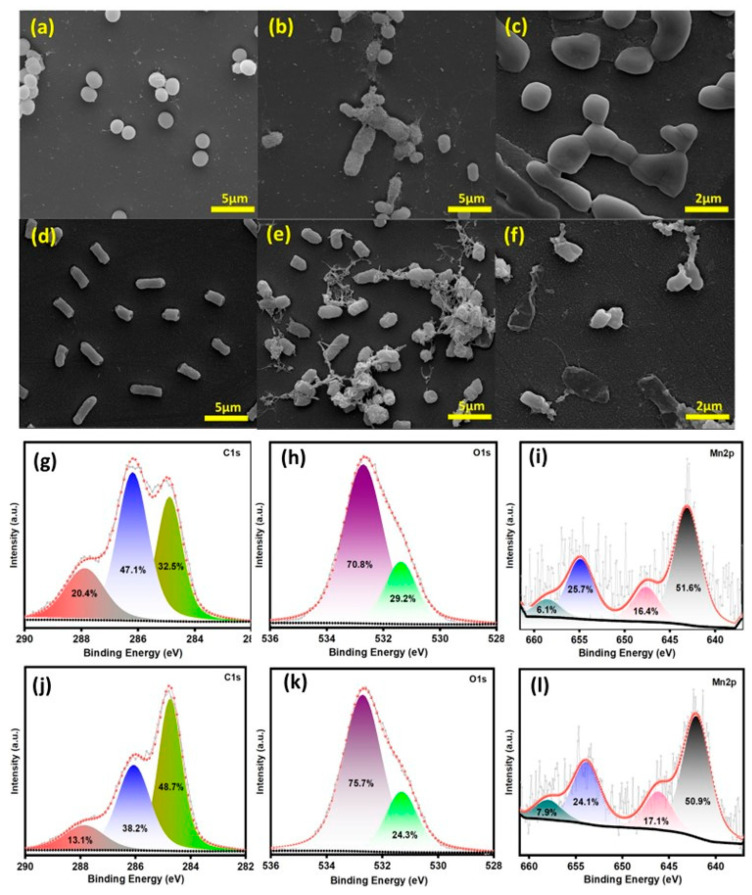
SEM images of (**a**–**c**) MDR *E.coli* biofilm and (**d**–**f**) MRSA biofilm inhibited by MnO_2_ NP exposure. The XPS spectrum (**g**–**i**) C1s, O1s, and Mn2p of MRSA and (**j**–**l**) C1s, O1s, and Mn2p of MDR *E. coli* after the biofilm Eradication.

In this study, SEM observations provide robust support that the interaction between MnO_2_ NPs and both bacterial strains exerted antibacterial activity by compromising the integrity of bacterial cell walls, highlighting the potent antibacterial properties of MnO_2_ NPs against the targeted bacterial species, encompassing both Gram-positive and Gram-negative bacteria. The bacterial membrane disruption, loss of cell integrity, and MnO_2_ NP–bacteria interaction, leading to cell death of the MRSA and MDR *E.coli*, were observed by SEM, which elucidated the cell death upon treating with MnO_2_ NPs. Figure 4 shows the spherical shape of MRSA and rod-shaped MDR *E. coli* before treatment with MnO_2_ NPs. After the treatment with MnO_2_ NPs, the bacterial surface exhibited an irregular and collapsed structure, indicating structural damage and severe rupture in the MDR *E. coli* and MRSA. MnO_2_ NPs induced oxidative stress via the production of reactive oxygen species (ROS) to damage bacterial membranes and cause cell lysis. The reduced activity against *P. aeruginosa* is attributed to its dense EPS architecture and elevated catalase activity. Nevertheless, EPR analysis demonstrated ROS levels exceeding PA’s antioxidant neutralization threshold, indicating that MnO_2_ remains active, but diffusion limitations restrict its efficacy. Future optimization may involve Ce^4+^ doping and polymer composite modification to enhance redox cycling or cationic surface functionalization to improve EPS penetration. Comparisons with ZnO, CuO, and Fe_3_O_4_ in the literature show similar or superior biocompatibility for MnO_2_, though direct experimental comparisons were not performed here.

Furthermore, superoxide radicals and surface oxygen vacancies over MnO_2_ with different phase structures were detected using Electron Paramagnetic Resonance (EPR) spectra. It is generally agreed that surface defects, including oxygen vacancies, could facilitate the formation of superoxide radicals by activating oxygen molecules with electrons. The results of EPR confirmed that more surface active oxygen species and oxygen vacancies existed in γ-MnO_2_, in agreement with the results of XPS. Figure 4g–l show the XPS results of MDR and MRSA treated by MnO_2_ samples. The functional groups and elemental compositions of C1s, O1s, and Mn2p were deconvoluted and compared with the literature. The XPS C1s (g) spectrum of the MDR exhibited the binding energies at 284.8, 286.1, and 288 eV groups, which correspond to the C-C/C-H (32.5 At.%), C-N/C-O (47.1 At.%), and C=O (20.4 At.%). The XPS C1s (j) spectrum of the MRSA exhibited binding energies at 284.8 eV, 286.1 eV, and 288 eV groups, which correspond to the C-C/C-H (48.7 At.%), C-N/C-O (38.2 At.%), and C=O (13.1 At.%) [31]. While this study establishes the potent initial antibiofilm activity of MnO_2_-NPs, their operational longevity under repeated or extended exposure conditions remains to be fully characterized. The retention of the Mn^4+^ valence state post-treatment, as evidenced by XPS analysis, indicates structural integrity and potential for sustained catalytic activity. However, dedicated recyclability assays (e.g., multiple antibiofilm cycles) and long-term storage stability tests were not performed. Future studies will systematically evaluate the durability of these nanocomposites to establish their viability for prolonged therapeutic applications.

The relative intensity of C-C/C-H groups in MDR is lower than that of MRSA, whereas the intensity of C-O/C-N and C=O groups in MRSA is higher than that of MDR. In the O1s deconvoluted spectrum (h) of MDR, there were two predominant peaks at 531.5 and 532.5 eV, which correspond to the O-C (29.2 At.%) and C=O (70.8 At.%) functional groups. In the case of MRSA, the peak intensity of O-C and C=O functional groups is 24.3 and 75.7 At.%, respectively (k). The variation of C-C, C-O, C-N, and C=O intensities between MRSA and MDR is due to the presence of hydrocarbon compounds with unsaturated fatty acids [32]. To determine the Mn valence state, the spin–orbit doublet comprising the main Mn 2p_3/2_ and Mn 2p_1/2_ peaks is located at ~642 and ~653 eV (Figure 4i,l). The spin energy separation of Mn after the bacterial treatment is 11.8 eV, slightly higher than that of pristine Mn. The 2p_3/2_ and Mn 2p_1/2_ peaks suggested the oxidation state of Mn^4+^ after the biofilm eradication.

The X-ray photoelectron spectroscopy (XPS) data for MnO_2_ before and after biofilm eradication further revealed important information about the surface chemistry changes that occur upon contact with multidrug-resistant (MDR) *E. coli* and MRSA biofilms. These changes are vital for understanding the mechanistic aspects of MnO_2_ NPs in eradicating stubborn biofilms associated with antibiotic-resistant bacteria. Upon understanding the XPS peak shifts of MnO_2_ NPs before and after biofilm treatment, the peaks at 642.3 and 654.2 eV (Figure 4g–i) display the existence of a highly oxidized surface dominated by Mn^4+^. After the treatment with bacteria, MRSA displayed XPS peak shifts to 643.1 and 654.9 eV, and for MDR *E. coli*, peaks at 642.1 and 653 eV in Figure 4j–l. These shifts reflect delicate changes in the Mn oxidation state and general chemical environments of the MnO_2_ NP surface, possibly resulting from redox interactions between biofilm and MnO_2_ NPs. The release of Mn^2+^ can interact with surface bacterial protein groups, resulting in enzyme inactivation and membrane function disruption, as shown in Figure 4b–e. Considering a negative zeta potential of MnO_2_ NPs as mentioned earlier, they should be repulsed by negatively charged bacterial membranes. However, MnO_2_ NPs with a negative charge still bind to the negatively charged bacterial membrane via stronger van der Waals, hydrophobic interactions, and other forces as mentioned earlier, resulting in the membrane ‘s permeability and damage of intracellular contents [20].

**Figure 5:**
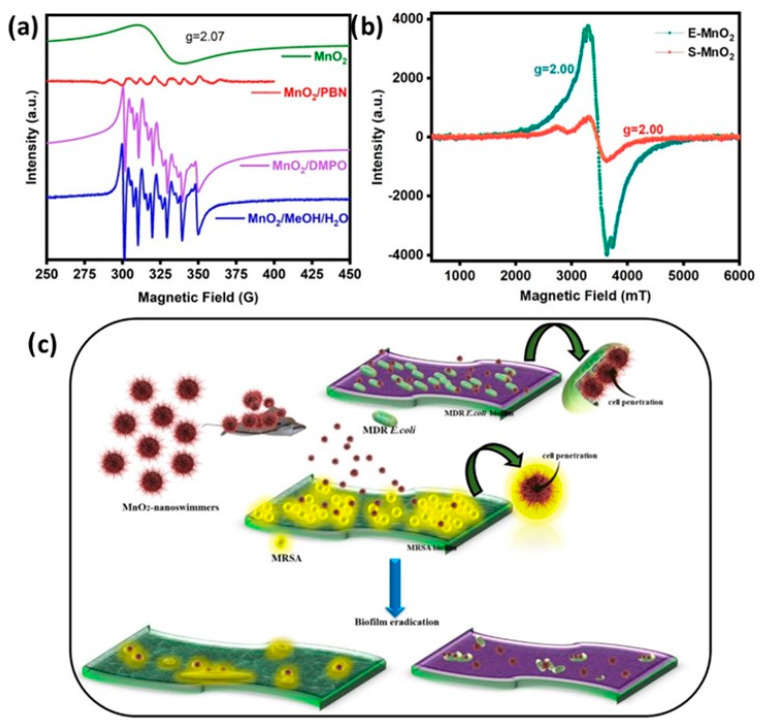
(**a**,**b**) EPR spectra of MnO_2_ before and after biofilm inhibition. (**c**) Illustration of the biofilm inhibition mechanism of MnO_2_ NPs against bacterial cells.

Physical interaction of MnO_2_ nanoparticles with bacteria, as discussed earlier, disrupts the membrane of Gram-positive MRSA and Gram-negative *E. coli*. This interaction increases permeability and leakage of cellular contents as displayed in Figure 4c–f. Treatment of MnO_2_ nanoparticles with MRSA shows a small increase in binding energy, which can be associated with oxidative transformation on the MnO_2_, indicating robust ROS formation and membrane oxidation. Upon treating MnO_2_ with MDR *E.coli*, a negligible reduction in binding energy was displayed, which could be due to the surface reduction and formation of Mn^3+^ via electron transfer from bacterial biofilm, corresponding with ROS-mediated degradation along with electron cycling. These changes indicate how MnO_2_ NPs’ catalytic behavior is dynamically controlled by bacterial biofilm type, allowing it to behave as an effective antimicrobial agent via surface oxidation (Figure 5a,b). The surface area and catalytic activity of MnO_2_ NPs boost its capability to interact with bacterial biofilms, eradicate complex systems, and disrupt membrane structures. Small MnO_2_ nanoparticles with high specific surface areas readily penetrate bacterial cells to induce damage to intracellular components by disrupting the cell membrane and subsequent loss of cellular contents. Released Mn^2+^ ions undergo contact with bacterial cell membranes, reducing the membrane dipole potential, altering the hydration state of the phospholipid head groups as well as the net surface charge of the membrane, causing local disruption of the membrane structure and increasing permeability.

The relative resistance of the PA biofilm to MnO_2_-mediated destruction deserves a brief discussion here. In brief, several strategies have been developed, including the use of bacteriophages, nanoparticles, enzymes, and natural products. These agents can invade the biofilm by various mechanisms and may be more effective when they are used in combination to inhibit *P. aeruginosa* biofilms. Lytic phage therapy is one of the important methods [33] because it leads to the rapid killing of their bacterial host cell [34,35]. Bacteriophage cocktails can easily penetrate and destroy the *P. aeruginosa* biofilm by synthesizing polysaccharide depolymerase [36] that decomposes the macromolecular carbohydrates of the bacterial envelope. Bacteriophages also generate peptidoglycan hydrolases called endolysins, antibacterial agents with high specific activity and a unique mode of action against bacteria, independent of antibiotic susceptibility patterns [35,37]. Bacteriophages induce the synthesis of quorum quenching (QQ) lactonase by genetic modification. This enzyme inhibits biofilm formation in *P. aeruginosa* by the hydrolysis of Acyl homoserine lactones and the inhibition of quorum-sensing activity [38]. However, after biofilm formation, phage treatment alone is often ineffective, and a combination of bacteriophages and selected antibiotics, e.g., ciprofloxacin and ceftazidime, effectively reduces bacterial density below that of the best single antibiotic treatment [39]. The combined use of bacteriophages and chestnut honey is effective in inhibiting *P. aeruginosa* PAO1 biofilms, and this pair might serve as a promising alternative for the topical treatment of wound infection [40]. Another interesting study is based on the combination of bacteriophage and chlorine disinfectants to destroy the PA biofilm. Chlorination treatment alone is ineffective because the EPS produced in the biofilm prevents the penetration of chlorine into the biofilm [41]. Xylitol enhances the bacteriophage function for the destruction of stable biofilm of *K. pneumonia* and *P. aeruginosa* [42]. Phage-inspired AuNP synthesis is effective against the biofilm-forming human bacterial pathogens [43]. In this context, MnO_2_ NPs can be combined with bacteriophases and/or the above-mentioned compounds to eradicate pre-existing films of *P. aeruginosa* and other bacterial biofilms, a subject of future endeavors. Although ROS-based nanomaterials are highly effective against pathogens with simpler or less protective biofilms, their activity is significantly attenuated in organisms such as *P. aeruginosa* that possess robust EPS matrices and strong intrinsic antioxidant defenses [44]. *Pseudomonas* EPS contains alginate, Pel (pellicle), and Psl (polysaccharide synthesis locus), which act as chemical and physical barriers that neutralize ROS and block antimicrobial penetration. Psl is a neutral, branched pentasaccharide composed of repeating units of D-mannose, D-glucose, and L-rhamnose. It is primarily involved in the early stages of biofilm development, anchoring the bacteria to surfaces and to each other. Psl is highly efficient at forming a protective, scaffold-like structure and helps the bacteria evade the immune system by inhibiting phagocytosis and preventing neutrophil ROS production [45]. Pel is a cationic (positively charged) polymer composed of 1–4 linked galactosamine and N-acetylgalactosamine. It is crucial for forming the “pellicle” (biofilm) at the air–liquid interface and maintaining the structural integrity of the mature biofilm. Due to its positive charge, Pel acts as a physical barrier that binds to and sequesters negatively charged components, including extracellular DNA (eDNA) and certain antibiotics (e.g., aminoglycosides like tobramycin). Both Pel and Psl act as a shield, preventing antibiotics, disinfectants, and host immune components (like reactive oxygen species—ROS) from reaching the bacteria inside the biofilm [44,45,46]). Figure 5c demonstrates the biofilm inhibition mechanism of MnO2 NPs against bacterial cells. The reduced activity against *P. aeruginosa* is attributed to its dense EPS architecture and elevated catalase activity. Nevertheless, EPR analysis demonstrated ROS levels exceeding PA’s antioxidant neutralization threshold, indicating that MnO_2_ remains active, but diffusion limitations restrict its efficacy. Future optimization may involve Ce^4+^ or Cu^2+^ doping to enhance redox cycling or cationic surface functionalization to improve EPS penetration. Comparisons with ZnO, CuO, and Fe_3_O_4_ in the literature show similar or superior biocompatibility for MnO_2_, though direct experimental comparisons were not performed here.

### 4.2. Cytotoxicity

The biocompatibility of MnO_2_ nanomaterials has been extensively documented in the literature. Multiple independent studies have demonstrated that MnO_2_ nanosheets and nanoparticles exhibit low cytotoxicity toward fibroblasts, keratinocytes, and macrophages at concentrations comparable to or higher than those used in our experiments. Recent reports indicate that MnO_2_ nanosheets exhibit high viability (>80–90%) in human dermal fibroblasts and epithelial cells, even at concentrations up to 100–200 µg/mL. Similarly, macrophage cell lines (RAW 264.7) exhibit minimal cytotoxicity and no significant LDH release, with MnO_2_ nanoparticles often displaying immunomodulatory rather than cytotoxic effects. Comprehensive reviews further confirm that MnO_2_ nanomaterials are generally biocompatible across a wide range of in vitro and in vivo models [47,48,49,50,51].

## 5. Conclusions

This study demonstrates a cost-effective and efficient method for synthesizing MnO_2_ nanoparticles (NPs) via an ultrasonic-assisted approach. The findings highlight the considerable potential of MnO_2_ NPs as potent antibiofilm agents capable of mitigating the growing threat of antimicrobial resistance. MnO_2_ exhibited outstanding biofilm inhibition efficiencies of over 98% against methicillin-resistant *Staphylococcus aureus* (MRSA) and multidrug-resistant *Escherichia coli*, but only 20% against *Pseudomonas aeruginosa*, achieving complete biofilm mass elimination within 24 h. The bacterial morphology and reactive oxygen species (ROS)-mediated mechanisms were investigated following biofilm eradication, revealing significant structural damage to bacterial cells. XPS analysis of MnO_2_ NPs following biofilm eradication confirmed the presence of Mn^4+^ species, indicating the high structural stability of the nanoparticles. The superior antibiofilm performance of MnO_2_ NPs could be attributed to their nanocatalytic activity and strong binding affinity toward both sensitive and drug-resistant pathogens. Future research should aim to extend the evaluation of their antibiofilm potential against a wider spectrum of clinically relevant pathogens and further elucidate the underlying molecular mechanisms, signaling pathways, and in vivo efficacy. MnO_2_ nanoparticles may be deployed in topical hydrogels for wound infections, sprayable nanoemulsions for skin and soft-tissue biofilms, implant coatings to prevent postoperative infections, and microneedle patches for localized delivery. Their biodegradability and safety profile support translational potential.

## Data Availability

Data available on request.
